# Flexibilisquinone, a New Anti-Inflammatory Quinone from the Cultured Soft Coral *Sinularia flexibilis*

**DOI:** 10.3390/molecules18078160

**Published:** 2013-07-10

**Authors:** Yu-Fang Lin, Chao-Ying Kuo, Zhi-Hong Wen, Yen-You Lin, Wei-Hsien Wang, Jui-Hsin Su, Jyh-Horng Sheu, Ping-Jyun Sung

**Affiliations:** 1 Department of Marine Biotechnology and Resources, National Sun Yat-sen University, Kaohsiung 804, Taiwan; E-Mails: yvonne6819@yahoo.com.tw (Y.-F.L.); wzh@mail.nsysu.edu.tw (Z.-H.W.); chas6119@gmail.com (Y.-Y.L.); whw@nmmba.gov.tw (W.-H.W.); 2 Graduate Institute of Marine Biotechnology, National Dong Hwa University, Pingtung 944, Taiwan; E-Mails: recall04729@hotmail.com (C.-Y.K.); x2219@nmmba.gov.tw (J.-H.S.); 3 National Museum of Marine Biology and Aquarium, Pingtung 944, Taiwan; 4 Division of Marine Biotechnology, Asia-Pacific Ocean Research Center, National Sun Yat-sen University, Kaohsiung 804, Taiwan; 5 Doctoral Degree Program in Marine Biotechnology, National Sun Yat-sen University and Academia Sinica, Kaohsiung 804, Taiwan; 6 Chinese Medicine Research and Development Center, China Medical University Hospital, Taichung 404, Taiwan; 7 Graduate Institute of Natural Products, Kaohsiung Medical University, Kaohsiung 807, Taiwan

**Keywords:** flexibilisquinone, cultured soft coral, *Sinularia flexibilis*, anti-inflammatory, iNOS, COX-2

## Abstract

A new quinone derivative, flexibilisquinone (**1**), was isolated from the cultured soft coral *Sinularia* *flexibilis*, originally distributed in the waters of Taiwan. The structure of quinone **1** was established by extensive spectroscopic methods, particularly 1D and 2D NMR experiments. In the *in vitro* anti-inflammatory effects test, quinone **1** was found to significantly inhibit the accumulation of the pro-inflammatory iNOS and COX-2 proteins of the LPS-stimulated RAW264.7 macrophage cells.

## 1. Introduction

**Figure 1:**
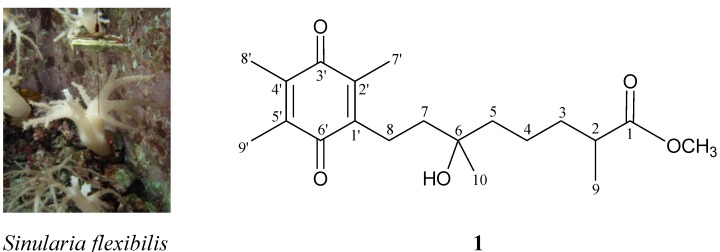
The cultured soft coral *S. flexibilis* and the structure of flexibilisquinone (**1**).

Soft corals belonging to the genus *Sinularia* are well-recognized as marine organisms containing various natural products that show interesting bioactivities [1,2]. Because all the corals are claimed to be threatened species, we therefore want to culture these interesting specimens as sources of potential natural products. In previous studies on the chemical constituents of cultured octocorals, a series of interesting secondary metabolites were obtained from *Erythropodium caribaeorum* [3], *Klyxum simplex* [4,5,6,7], *Lobophytum crassum* [8], *Sarcophyton trocheliophorum* [9], *Sinularia flexibilis* [10], *Sinularia leptoclados* [11], *Briareum excavatum* [12,13,14,15,16,17,18,19,20] and *Briareum* sp. [21]. Two novel metabolites, pseudoalteromones A and B were obtained from a marine bacterium *Pseudoalteromonas* sp. CGH2XX, a bacterium originally isolated from a cultured soft coral *Lobophytum crassum* [22,23]. During the course of our further investigation on new natural substances from the cultured soft coral *Sinularia flexibilis* (Figure 1), a new quinone derivative, flexibilisquinone (**1**), has been isolated.

## 2. Results and Discussion

Flexibilisquinone (**1**) was isolated as a yellow oil that gave an [M + Na]^+^ ion peak at *m/z* 373.1988 in the HRESIMS, corresponding to a molecular formula C_20_H_30_O_5_ (calcd. for C_20_H_30_O_5_Na, 373.1991) requiring six degrees of unsaturation. The IR absorptions bands at 3490, 1736, 1680 and 1642 cm^–^^1^ were characteristic of hydroxy, ester and quinone moieties, and the latter deduction was further supported by the UV absorptions at λ_max_ 262 (log ε 3.8) and 267 (log ε 3.8) nm. The ^13^C-NMR data of **1** (Table 1) showed the presence of 20 carbon signals, which were identified by the assistance of a DEPT spectrum as six methyls, five sp^3^ methylenes, an sp^3^ methine, an sp^3^ quaternary carbon and seven sp^2^ quaternary carbons including three carbonyls. The ^1^H-NMR spectrum of **1** showed signals of a methoxy group (*δ*_H_ 3.68, 3H, s), three vinyl methyls (*δ*_H_ 2.03, 2.01, 2.01, each 3H × s), a methyl doublet (*δ*_H_ 1.17, 3H, d, *J* = 7.2 Hz), a methyl singlet (*δ*_H_ 1.22, 3H, s), five pairs of aliphatic methylene protons (*δ*_H_ 1.67, 1H, m; 1.40, 1H, m; 1.45, 1H, m; 1.38, 1H, m; 1.74–1.39, 4H, m; 2.53, 2H, m) and an aliphatic methine proton (*δ*_H_ 2.48, 1H, m).

**Table 1:** ^1^H- (400 MHz, CDCl_3_) and ^13^C- (100 MHz, CDCl_3_) NMR data, ^1^H–^1^H COSY and HMBC correlations for quinone **1**.

Position	*δ*_Η_ (*J* in Hz)	*δ*_C_, Mult.	^1^H–^1^H COSY	HMBC (H→C)
1		177.2, C		
2	2.48 m	39.4, CH	H_2_-3, H_3_-9	C-1, -3, -4, -9
3	1.67 m; 1.40 m	34.2, CH_2_	H-2, H_2_-4	C-1, -2, -4, -5, -9
4	1.45 m; 1.38 m	21.6, CH_2_	H_2_-3, H_2_-5	C-2, -3, -5, -6
5	1.74–1.39 m	41.7, CH_2_	H_2_-4	C-3, -4, -6, -7, -10
6		72.5, C		
7	1.74–1.39 m	40.2, CH_2_	H_2_-8	C-5, -6, -8, -10, -1'
8	2.53 m	21.3, CH_2_	H_2_-7	C-6, -7, -1', -2', -6'
9	1.17 d (7.2)	17.1, CH_3_	H-2	C-1, -2, -3
10	1.22 s	26.6, CH_3_		C-5, -6, -7
1'		144.3, C		
2'		140.2, C		
3'		187.6, C		
4'		140.4, C *^a^*		
5'		140.6, C *^a^*		
6'		187.2, C		
7'	2.03 s	12.0, CH_3_		C-1', -2', -3'
8'	2.01 s	12.3, CH_3_*^b^*		C-3', -4', -5'
9'	2.01 s	12.4, CH_3_*^b^*		C-4', -5', -6'
1-OCH_3_	3.68 s	51.5, CH_3_		C-1

*^a,b^* Data exchangeable.

**Figure 2:**
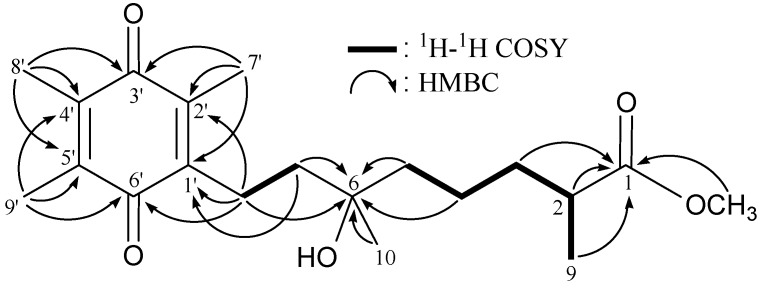
The ^1^H–^1^H COSY and selective key HMBC correlations (protons→quaternary carbons) of **1**.

From the ^1^H–^1^H COSY spectrum of **1** (Table 1 and Figure 2), it was possible to establish the separate spin systems that map out the proton sequences from H-2/H_2_-3/H_2_-4/H_2_-5, H_2_-7/H_2_-8 and H-2/H_3_-9. These data together with the key HMBC correlations between protons and quaternary carbons (Table 1 and Figure 2), such as H-2, H_2_-3/C-1 and H_2_-4, H_2_-5, H_2_-7, H_2_-8/C-6, permitted the elucidation of the straight carbon skeleton of the side chain. The methyls at C-2 and C-6 were confirmed by the HMBC correlations between H_3_-9/C-1, -2, -3 and H_3_-10/C-5, -6, -7, respectively. The monoterpenoid side chain which is fused to the quinone moiety at C-1', was elucidated by the HMBC correlations between H_2_-7, H_2_-8/C-1' and H_2_-8/C-2', -6'. The C-7', C-8' and C-9' vinyl methyls at C-2', C-4' and C-5' were established by the HMBC correlations between H_3_-7'/C-1', -2', -3'; H_3_-8'/C-3', -4', -5'; and H_3_-9'/C-4', -5', -6', respectively. The methoxy group at C-1 was elucidated by the HMBC correlations between methyl group at *δ*_H_ 3.68 (3H, s) and an ester carbonyl at *δ*_C_ 177.2 (C). Based on the above findings, the structure of **1** was established unambiguously.

The spectral data of **1** were in full agreement with those of a known quinone analogue, sarcophytonone, which was isolated from a Chinese soft coral *Sarcophyton crassocaule* [24]. However, the optical rotation value of **1** {[α${]}_{\text{D}}^{23}$ −19.6 (*c* 0.78, CHCl_3_)} [25] was substantially different from that of sarcophytonone ([α${]}_{\text{D}}^{25}$ +5.82 (*c* 0.40, CHCl_3_)), indicating that quinone **1** is an enantiomer of sarcophytonone, by comparison the structure of **1** with that of sarcophytonone.

**Figure 3:**
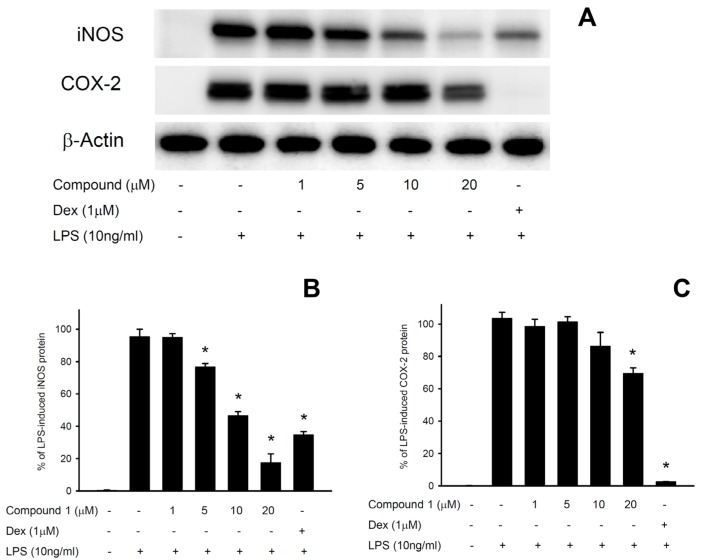
Effect of compound **1** on pro-inflammatory iNOS and COX-2 protein expression in LPS-stimulated murine macrophage cell line RAW264.7. (**A**) Western blots for iNOS, COX-2 and β-actin proteins from macrophage cells; (**B**) relative density of iNOS immunoblot; (**C**) relative density of COX-2 immunoblot. The relative intensity of the LPS-stimulated group was taken to be 100%. Band intensities were quantified by densitometry and are indicated as the percent change relative to that of the LPS-stimulated group. Compound **1** and dexamethasone (Dex) significantly inhibited LPS-induced iNOS and COX-2 protein expression in macrophage. The experiment was repeated three times. (* *p* < 0.05, significantly different from the LPS-stimulated group).

The dose inhibition of compound **1** on LPS-induced pro-inflammatory iNOS (inducible nitric oxide synthase) and COX-2 (cyclooxygenase-2) proteins expression was evaluated by western blot analysis (Figure 3). The result clearly depicts an up-regulation of iNOS and COX-2 proteins in LPS-stimulated murine macrophage cell line. Both iNOS and COX-2 were significantly inhibited by compound **1** at 5–20 µM and 20 µM, respectively. Only the vehicle (DMSO) did not induce up-regulation of iNOS and COX-2 protein expression. Furthermore, compound **1** (1–20 µM) did not induced obviously cytotoxicity in macrophage cells, as determined through Trypan blue staining.

## 3. Experimental

### 3.1. General

Optical rotations were measured with a JascoP1010 digital polarimeter (Japan Spectroscopic Corporation, Tokyo, Japan). Infrared spectra were obtained on a Varian Diglab FTS 1000 FT-IR spectrophotometer (Varian Inc., Palo Alto, CA, USA). UV spectra were recorded on a Hitachi U-3210 UV spectrophotometer (Hitachi Ltd. Tokyo, Japan). NMR spectra were recorded on a Varian Mercury Plus 400 NMR spectrometer (Varian Inc.) at 400 MHz for ^1^H and 100 MHz for ^13^C in CDCl_3_ at 25 °C. ESIMS and HRESIMS data were recorded on Bruker APEX II mass spectrometer (Bruker, Bremen, Germany). Column chromatography was performed on silica gel (230–400 mesh, Merck, Darmstadt, Germany). TLC was carried out on precoated Kieselgel 60 F_254_ (0.25 mm, Merck) and spots were visualized by spraying with 10% H_2_SO_4_ solution followed by heating. Normal phase HPLC (NP-HPLC) was performed using a system comprised of a Hitachi L-7110 pump (Hitachi Ltd. Tokyo, Japan) and a Rheodyne 7725 injection port (Rheodyne LLC. Rohnert Park, CA, USA). A normal phase column (Supelco Ascentis^®^ Si Cat #:581515-U, 25 cm × 21.2 mm, 5 µm, Sigma-Aldrich, St. Louis, MO, USA) was used for NP-HPLC.

### 3.2. Animal Material

Specimens of the cultured soft coral *Sinularia flexibilis* (specimen no. CSC-1) were collected by hand in a 80 ton cultivation tank located in the National Museum of Marine Biology and Aquarium (NMMBA), Taiwan, in July 2006 and stored in a freezer (−20 °C) until extraction. A voucher specimen was deposited in the Department of Marine Biotechnology and Resources, National Sun Yat-sen University, Kaohsiung, Taiwan.

### 3.3. Extraction and Isolation

The freeze-dried and minced material of the cultured soft coral *Sinularia* *flexibilis* (wet weight 1.5 kg) was extracted exhaustively with ethanol (EtOH) at 25 °C (1L × 6). The EtOH extract was filtered and concentrated under reduced pressure. The residue was partitioned between dichloromethane (CH_2_Cl_2_) and H_2_O. The CH_2_Cl_2_-soluble fraction was concentrated and the residue was chromatographed on Si gel by column chromatography and eluted with ethyl acetate (EtOAc) in *n*-hexane (0−100%, gradient) to yield 25 fractions. Fraction 15, eluted with EtOAc-*n*-hexane (1:2), was further purified by NP-HPLC using EtOAc-*n*-hexane (1:5) to yield **1** (7.8 mg, 0.00052%). 

*Flexibilisquinone* (**1**): [α${]}_{\text{D}}^{23}$ −19.6 (*c* 0.78, CHCl_3_); UV (MeOH) λ_max_ (log ε) 262 (3.8), 267 (3.8) nm; IR (neat) ν_max_ 3490, 1736, 1680, 1642 cm^−1^; ^1^H- (CDCl_3_, 400 MHz) and ^13^C- (CDCl_3_, 100 MHz) NMR data, see Table 1; ESIMS *m/z* 373 [M + Na]^+^; HRESIMS: *m/z* 373.1988 (calcd for C_20_H_30_O_5_Na, 373.1991).

### 3.4. *In Vitro* Anti-Inflammatory Assay

Murine macrophage (RAW264.7) cell line was purchased from ATCC. *In vitro* anti-inflammatory activity of compound **1** was measured by examining the inhibition of lipopolysaccharide (LPS)-induced up-regulation of pro-inflammatory iNOS (inducible nitric oxide synthase) and COX-2 (cyclooxygenase-2) proteins expression in macrophage cells using western blotting analysis [26,27,28]. Briefly, inflammation in macrophages was induced by incubating them for 16 h in a medium containing only LPS (10 ng/mL) without compounds. For anti-inflammatory activity assay, compound **1** (1, 5, 10 or 20 µM) or dexamethasone (Dex; 1 µM) were added the cells 10 min before LPS challenge. The cells were then for western blot analysis. The immunoreactivity data are calculated with respect to the average optical density of the corresponding LPS-stimulated group. For statistical analysis, the data were analyzed by a one-way analysis of variance (ANOVA), followed by the Student-Newman-Keuls *post hoc* test for multiple comparisons. A significant difference was defined as a *P* value of <0.05.

## 4. Conclusions

Octocorals have been well-recognized as an important source of potential medicinal-use agents. However, because of the corals are claimed to be threatened species and most of the compounds from octocorals are difficult to obtain by chemical methods, bioactive compounds from cultured soft corals will play an important role in this field. Our further studies on the chemical constituents of a cultured soft coral *Sinularia flexibilis* grown in the culture tanks with a flow-through sea water system located in the National Museum of Marine Biology and Aquarium, Taiwan for the extraction of additional natural products in order to establish a stable supply of bioactive material, have led to the isolation of a new quinone derivative, flexibilisquinone (**1**), and this compound was found to significantly inhibit the accumulation of the pro-inflammatory iNOS protein of the LPS-stimulated RAW264.7 macrophage cells, suggesting that quinone **1** is worthy of further biomedical investigation.
